# Treatment of Schatzker Type II-VI Tibial Plateau Fractures by Means of Syndesmotaxis Using an Ilizarov External Fixator and Postoperative CT Evaluation

**DOI:** 10.7759/cureus.12680

**Published:** 2021-01-13

**Authors:** Stamatios A Papadakis, Dimitrios Pallis, Margarita-Michaela Ampadiotaki, Georgios Gourtzelidis, Konstantinos Kateros, George Macheras

**Affiliations:** 1 B' Orthopaedic Department, KAT General Hospital of Attica, Athens, GRC; 2 A' Orthopaedic Department, General Hospital G.Gennimatas, Athens, GRC; 3 D' Orthopaedic Department, KAT General Hospital of Attica, Athens, GRC

**Keywords:** tibial plateau fractures, ilizarov fixator, computed tomography

## Abstract

Introduction

Tibial plateau fractures are more common in young patients following high-energy trauma. In this study, we aim to evaluate the articular surface reduction quality by means of postoperative computer tomography (CT) in Schatzker type II-VI tibial plateau fractures treated with an Ilizarov frame.

Materials and methods

This case series study included 45 patients with a mean age of 39.5 years (range: 18 to 65 years) with a Schatzker type II-VI tibial plateau fracture. The surgical technique was a mini-open reduction of the articular surface impaction followed by application of an Ilizarov circular frame with knee bridging. Pre- and postoperative CT scan evaluation was performed in all of the patients. Outcomes were measured using the American Knee Society Score (AKSS). Mean outpatient follow-up was of at least 12 months (range: 12 to 21 months). Mean time for fracture consolidation was 15.5 weeks (range: 13 to 19 weeks). According to the degree of postoperative articular surface impaction, patients were grouped as follows: 11 had less than 2 mm of depression, 27 had 2 to 4 mm of depression, and 7 over 4 mm of depression.

Results

Patients with articular surface impaction of more than 4 mm presented statistically significant lower values of AKSS compared to those with impaction of lower than 2 mm (p<0.001 ) and 2-4 mm (p<0.001). Patients with joint alignment equal to or more than 5^°^ presented statistically significant lower values of AKSS compared to those with lower than 5^°^.

Conclusions

Schatzker type II-VI tibial plateau fractures may be treated successfully with mini-open reduction and the application of an Ilizarov frame. The increase of articular surface impaction by 1 mm causes reduction of AKSS by 15 units. Patients with joint alignment equal to or more than 5^°^ present lower values of AKSS. The preoperative CT scan is important and useful in planning the surgical intervention no matter the classification system is used.

## Introduction

Tibial plateau fractures are more common in young patients following high-energy trauma. These fractures in the elderly with osteoporotic bone are caused by low-energy fall. Fractures of tibial plateau are caused by direct axial compression combined with a varus or valgus force. These fractures are usually associated with soft tissue injuries. The severity of soft tissue injuries should be evaluated properly and not be underestimated. Associated vascular injuries and compartment syndrome are not uncommon in complex tibial plateau fractures [1].

The anatomy of the tibial plateau is complex and should be keeping in mind during the reduction. The shape of the tibial plateau is asymmetric and has a direct role in the biomechanics of the tibiofemoral joint. The position of the knee at impact and the characteristics of the force such as the direction and the magnitude determine the injury pattern and the damage of soft tissue envelope [2].

 Injury pattern, articular surface impaction, soft tissue condition, condylar dissociation, and extension of fracture line in metaphysis should be considered prior to the treatment method [2]. Ilizarov external fixator provides adequate stabilization of these fractures and restores the articular surface and the mechanical axis without compromising the condition of the soft tissue envelope. Restoration of articular surface and of mechanical axis correlates most with a satisfactory clinical outcome [3].

## Materials and methods

This prospective case series study included 45 patients, with a mean age of 39.5 years (range: 18 to 65 years), and the fracture types were classified according to the Schatzker classification [4]. Inclusion criteria were the presence of complex Schatzker type II-VI intra-articular proximal tibia fractures, patient age over 18 years, and the ability to walk without assistance before injury. Patients with bilateral tibial plateau fracture, those with an associated ipsilateral femoral fracture, and those lost to follow-up were excluded from the study. The study was approved by the Scientific Committee of our institution. The majority of cases were of closed injuries, except two cases (Gustilo-Anderson type 3a and type 2). In all of the cases, prophylactic antibiotics were administered intravenously according to protocol. Two patients with open fractures were treated with debridement and irrigation (Table 1). Closed fractures were operated with an average of four days’ delay (range: 3 to 7 days) in order to allow amelioration of soft tissue damage. Pre- and postoperatively, a CT scan of the injured knee was performed in order to evaluate the fracture pattern, the reduction of the articular surface, and the restoration of the mechanical axis. Follow-up duration ranged from 12 to 21 months. Outcome was measured using the American Knee Society Score (AKSS) at the 12 months’ assessment.

**Table 1:** Data of patients

Case Number	Age	Schatzker type	Open fracture	Preoperative step (mm)	Joint alignment (0^°^)	Articular surface impaction (mm)	AKSS total
1	18	VI	No	28	4 Varus	< 2	191
2	22	V	No	13	9 Varus	3	182
3	29	VI	No	16	12 Valgus	4	141
4	23	VI	No	9	7 Valgus	2	169
5	39	V	No	11	5 Varus	4	149
6	36	V	No	14	3 Valgus	2	183
7	32	VI	Gustilo type IIIA	11	7 Varus	5	147
8	45	III	No	12	7 Varus	3	180
9	55	II	No	10	3 Varus	3	171
10	36	V	No	9	4 Varus	3	184
11	37	V	No	7	3 Varus	<2	184
12	51	VI	No	19	3 Valgus	5	126
13	24	VI	No	33	3 Valgus	4	180
14	45	V	Gustilo type II	12	9 Varus	5	129
15	42	VI	No	17	3 Varus	5	123
16	48	VI	No	13	4 Varus	6	119
17	51	VI	No	11	2 Valgus	<2	183
18	33	V	No	9	2 Varus	<2	190
19	42	VI	No	15	8 Valgus	6	119
20	44	V	No	9	4 Varus	3	171
21	46	II	No	7	2 Varus	<2	184
22	36	V	No	8	2 Varus	2	172
23	39	VI	No	7	2 Varus	<2	177
24	35	VI	No	13	5 Valgus	5	126
25	47	V	No	9	2 Varus	<2	177
26	41	V	No	14	2 Varus	2	172
27	29	V	No	9	3 Varus	3	164
28	43	V	No	11	3 Varus	3	170
29	36	V	No	13	2 Varus	3	166
30	28	V	No	8	5 Varus	<2	185
31	36	V	No	9	2 Valgus	<2	192
32	43	VI	No	12	4 Varus	4	164
33	39	V	No	9	7 Varus	3	160
34	57	V	No	8	4 Valgus	3	172
35	19	VI	No	11	3 Varus	<2	192
36	33	V	No	9	2 Varus	3	177
37	34	V	No	7	4 Varus	3	176
38	37	V	No	12	2 Varus	4	156
39	50	V	No	9	5 Varus	3	164
40	49	V	No	9	3 Varus	<2	185
41	65	VI	No	8	5 Varus	2	171
42	36	V	No	15	3 Varus	3	170
43	47	VI	No	9	2 Valgus	3	184
44	59	III	No	8	2 Varus	2	177
45	43	II	No	11	3 Valgus	3	176

 Surgical technique

**Figure 1:**
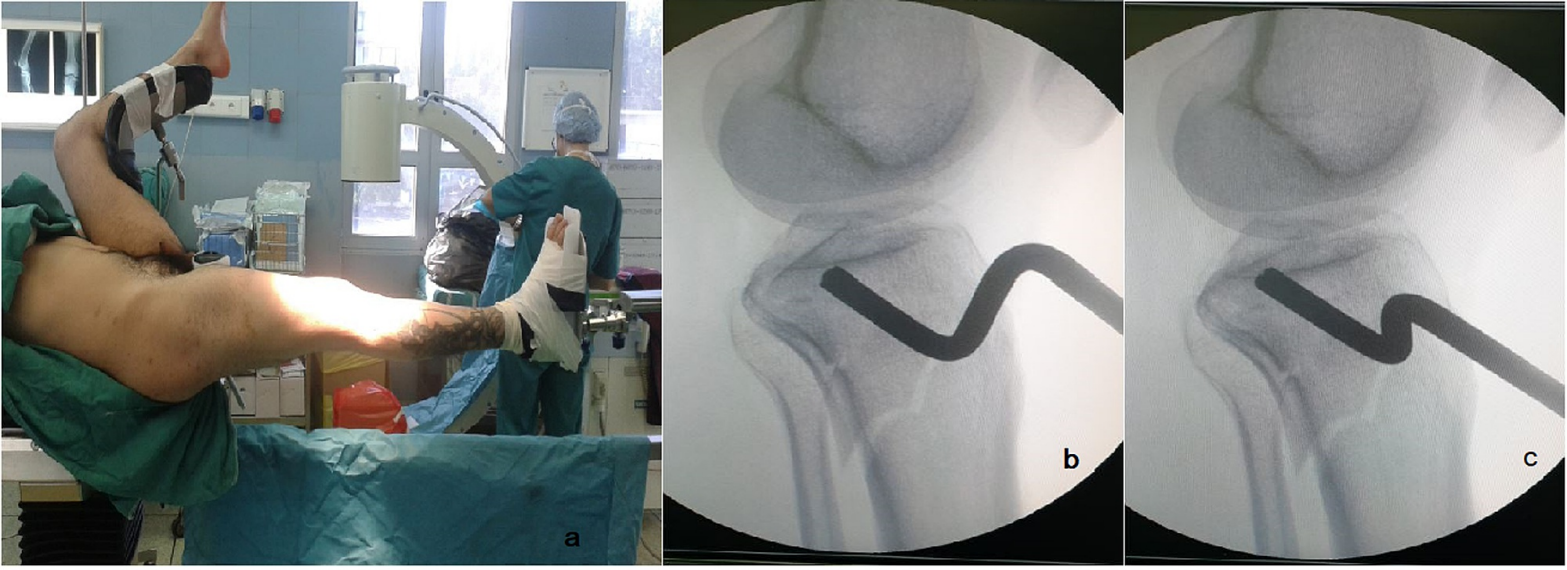
(a) Photo of the patient position on the traction table. (b, c) Intraoperative fluoroscopy images showing a depression of the articular surface and its reduction using an elevator.

All patients were positioned supine on a traction table with the knee extended to assist in the reduction (Figure 1a). A traction table contributes 360° visualization using an image intensifier and allows the use of a circular ring fixator without impingement on the table [5]. The reduction is achieved through ligamentotaxis. Ligaments can transfer load from bone to bone, lengthwise the axis that they are acting [6]. The soft tissue envelope transfers load to the bony fragments, and through traction, alignment of the joint is achieved by indirect reduction (Table 2).

**Table 2:** Structures around the knee and their action. [7]

Structures	Tight in flexion	Tight In extension
Anterior cruciate ligament	Anteromedial bundle	Anterolateral bundle
Posterior cruciate ligament	Posterolateral bundle	Posteromedial bundle
Lateral collateral ligament	Lax in flexion	Tight in extension
Medial collateral ligament	-	Tight in extension and valgus
Quadriceps femoris muscle	Tight in flexion	-
Semitendinosus muscle	-	Tight in extension and external rotation
Semimembranosus muscle	-	Tight in extension and external rotation
Sartorius muscle	-	Tight in extension and external rotation
Popliteus muscle		Tight in extension and external rotation
Iliotibial band	-	Tight in extension and internal rotation
Gastrocnemius muscle	-	Tight in extension
Biceps femoris muscle	-	Tight in extension and internal rotation
Gracilis muscle	-	Tight in extension and external rotation
Articular capsule	-	Tight in extension

In this way, bony fragments attached to the soft tissue are positioned back in place. Furthermore, the joint capsule and the attachment of muscles and tendons masses surrounding the knee joint could put pressure on bony fragments and thus contribute to the reduction. The intensity of the traction for the reduction of the articular alignment is controlled through fluoroscopic imaging. However, impacted articular fragments remain unreduced. In these cases with inadequate reduction, a 1.5- to 3-cm incision is performed over the anterolateral aspect of the tibial metaphysis. Through this opening, an elevator is used under fluoroscopy for the elevation and reduction of the articular surface (Figures 1b, 1c). Allografts are used in order to fill the remaining osseous gap. Two cannulated screws are inserted below the articular surface in order to restore and maintain the reduction, the joint expansion, and the position of the allografts. An Ilizarov frame bridging the knee is placed under traction. Bridging is used to preserve the reduction and to maintain the joint extension and stability. The proximal tibial wires are positioned at least 1 cm below the articular surface, avoiding a possible septic arthritis due to pin track infection [7]. Knee bridging was applied in all of the cases and lasts up to six weeks.

We do not use arthroscopy to evaluate the articular reduction as we believe that the pressure of the arthroscopy fluids may lead to misalignment or displacement of the articular bony fragments. Also, there may be a risk factor of compartment syndrome that could compromise negatively the soft tissue envelope.

Statistical analysis

Data were expressed as means for quantitative variables and as percentages for categorical variables. The Kolmogorov-Smirnov test was utilized for normality analysis of the quantitative variables. Correlation between functional variables was examined using Spearman’s correlation coefficient.

Comparisons between different categories of qualitative variables in relation to AKSS score were performed using the independent samples t-test and one-way analysis of variance model (pairwise comparisons examined using Bonferroni correction).

All variables whether or not they demonstrated significant associations with outcome in unifactorial analysis were included in the multiple linear regression models, and the enter method was used to explore the independent predictors of AKSS score.

All assumptions of linear regression analysis (homoscedasticity, linearity, normality, and independence of error terms, as well as multicollinearity of independent variables) were examined.

All tests were two-sided, and the statistical significance was set at p<0.05. All analyses were carried out using SPSS Version 21.00 (IBM Corporation, Armonk, NY, USA).

## Results

All fractures healed, and the mean time for fracture consolidation was 15.5 weeks (range: 13 to 19 weeks). According to postoperative CT scan and degree of postoperative articular surface impaction, patients were grouped as follows: 11 (24.4%) had less than 2 mm of impaction, 27 (60%) had 2 to 4 mm of impaction, and 7 (15, 5%) over 4 mm of impaction. The AKSS was good or excellent in 21 patients and poor or fair in 10 patients. The AKSS function score was good or excellent in 29 patients and poor or fair in 16 (Table 3).

**Table 3:** Patients knee scores AKSS, American Knee Society Score

AKSS knee score category				AKSS function score category			
Poor (<60)	Fair (60-69)	Good (70-79)	Excellent (80-100)	Poor (<60)	Fair (60-69)	Good (70-79)	Excellent (80-100)
3	7	14	21	5	11	8	21
6.6%	15.5%	31.1%	46.6%	11.1%	24.4%	17.7%	46.6%

Preoperative step (p=0.018) and joint alignment (p=0.004) were moderate and negatively correlated with AKSS, and articular surface impaction (p<0.001) was high and negatively correlated with AKSS (Table 4).

**Table 4:** Demographic and clinical characteristics Preoperative step (r= -0,352; p=0.018) and joint alignment (r= -0,422; p=0.004) were moderate and negatively correlated with AKSS score, and articular surface impaction (r= -0,892; p<0.001) was very high and negatively correlated with AKSS score. AKSS, American Knee Society Score

Characteristics	
Age	39.5 ( 18-65)
Schatzker type II/III/V/VI, n (%)	5 (11.1)/24 (53.3)/16 (5.6)
Open fracture, no/yes, n (%)	43 (95.6)/2 (4.4)
Preoperative articular impaction (mm), mean (min-max)	11.6 (7-33)
Joint alignment (grade), mean (range)	4.0 (2-12)
Joint alignment (type), varus/valgus, n (%)	33 (73.3)/12 (26.7)
Joint alignment (grade), <5/≥5	32 (71.1)/13 (28.9)
Articular surface impaction (mm), mean (range)	3.1 ( 2-6)
Articular surface impaction (mm), <2/2-4/>4, n (%)	11 (24.4)/27 (60.0)/7 (15.6)
AKSS, mean (range)	166.4 (119-192)

A multiple regression model with the enter method was employed to examine the contribution of demographic and functional variables to AKSS score. Only the amount of articular surface impaction (p<0.001) was a statistically significant prognostic factor of AKSS score (Table 5).

**Table 5:** Multifactorial linear regression of AKSS score The amount of articular surface impaction (beta coefficient ± SE: -14.96 ± 1.65; R2 = 80%; p<0.001) was a statistically significant prognostic factor of AKSS score. Based on the very high value of R^2^ of articular surface impaction variable in the multiple linear regression models, we can assume that the articular surface impaction is the most important factor that affects the functional status of a patient. SE, standard error; AKSS, American Knee Society Score

	Reference category	R^2^	Beta	SE	p-Value
Age	---	<0.01	-0.23	0.16	0.149
Schatzker type	II-III-V	<0.01	-4.47	3.57	0.218
Preoperative step (mm)	---	<0.01	0.21	0.38	0.588
Joint alignment (grade)	---	0.01	-0.78	0.71	0.278
Joint alignment (valgus)	Varus	<0.01	2.33	3.52	0.512
Articular surface impaction (mm)	---	0.80	-14.96	1.65	<0.001

Patients with Schatzker type VI presented statistically significant lower values of AKSS score compared to those with type II-IV (p=0.009) and type V (p<0.05). Patients with articular surface impaction of more than 4 mm presented statistically significant lower values of AKSS compared to those with impaction of lower than 2 mm (p<0.001 ) and 2-4 mm (p<0.001) (Table 6).

**Table 6:** Correlation between AKSS and qualitative functional variables Patients with Schatzker type VI presented statistically significant lower values of AKSS compared to type II-IV (p=0.009) and type V (p=0.054). Patients with joint alignment equal to or more than 5° presented statistically significant lower values of AKSS compared to those with lower than 5° (p=0.022). Patients with articular surface impaction of more than 4 mm presented statistically significant lower values of AKSS compared to those with lower than 2 mm (p<0.001) and 2-4 mm (p<0.001). ^a^p=0.009 vs. VI;^ b^p=0.054 vs. VI; ^c^p<0.001 vs. >4; ^d^p<0.001 vs. II-IV AKSS, American Knee Society Score

		n	AKSS score	p-Value
Schatzker type	II-III	5	177.6±4.8^a^	0.008
	V	24	172.0±14.1^b^	
	VI	16	154.4±26.1	
Joint alignment (type)	Varus	33	167.8±18.2	0.541
	Valgus	12	162.6±26.6	
Joint alignment ( grade)	<5	32	170.8±18.5	0.022
	≥5	13	155.5±22.2	
Articular surface impaction (mm)	<2	11	184.7±5.1^c,d^	<0.001
	2-4	27	169.1±10.8^c^	
	>4	7	127.0±9.6	

**Figure 2:**
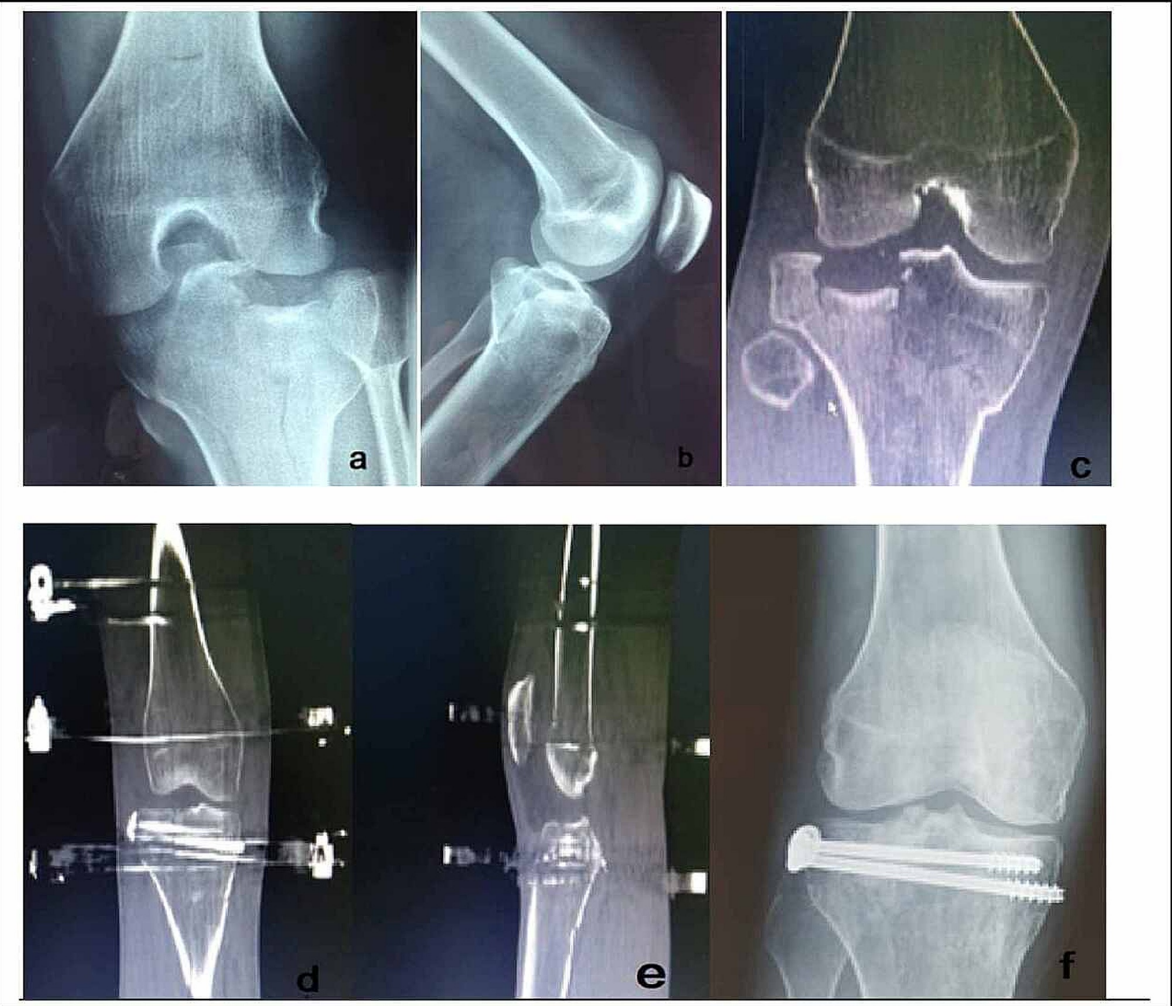
An 18-year-old man with a displaced bicondylar tibial plateau fracture. (a, b) Anteroposterior and lateral preoperative X-ray (c) Preoperative axial CT scan of the knee joint. (d) Postoperative CT scan (axial view) following mini-open reduction of the articular surface and application of an Ilizarov frame with knee bridging. (e) Postoperative sagittal CT scan view of the same patient. (f) Anteroposterior X-ray 5 months postoperatively.

Furthermore, patients with joint alignment equal to or more than 5° presented statistically significant lower values of AKSS compared to those with lower than 5° (p=0.022) (Table 6). All fractures healed, and there were no complications such as deep venous thrombosis, malunion, non-union (septic or no), osteomyelitis, and systemic complications (Figure 2).

**Figure 3:**
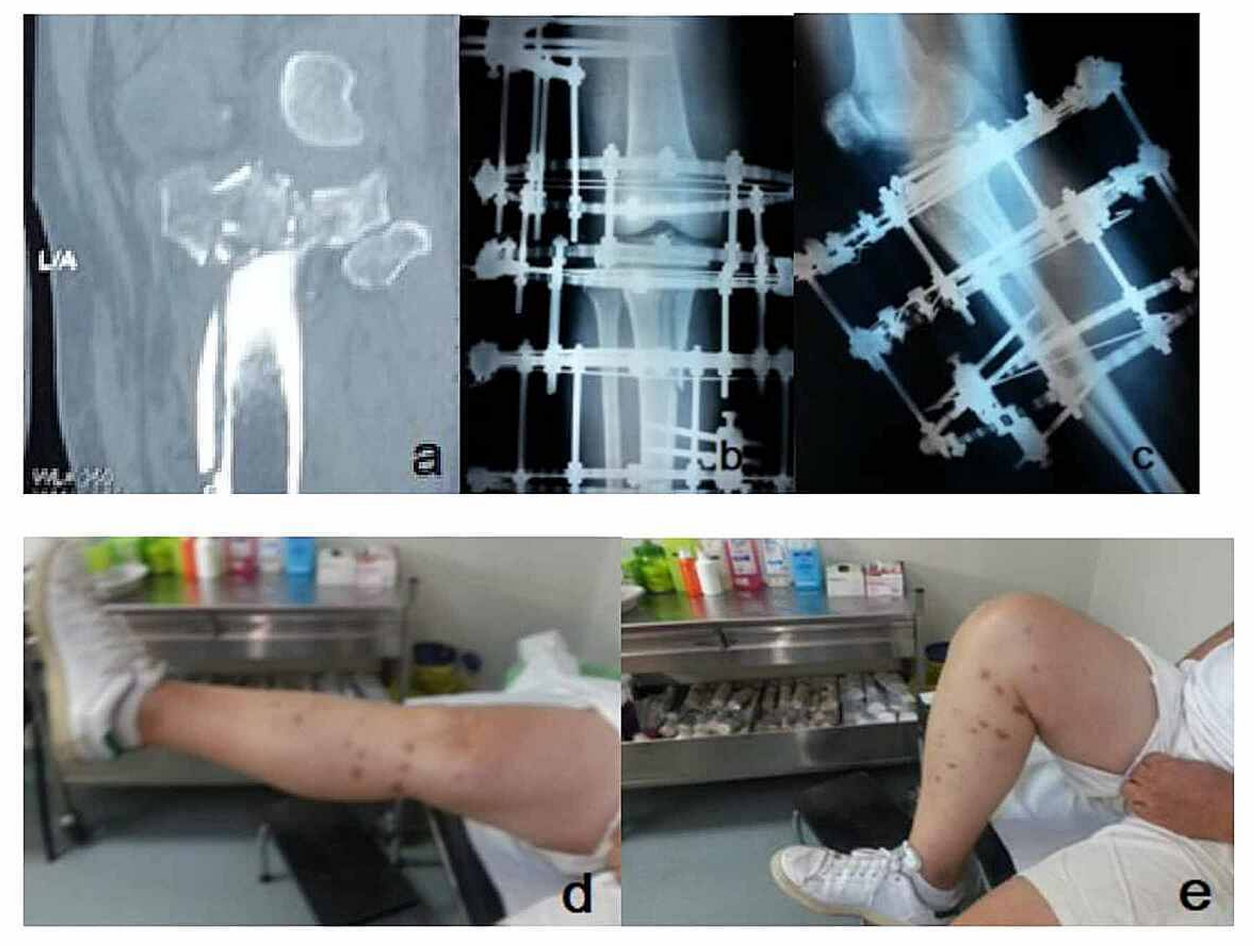
A 65-year-old man with a Schatzker type VI tibial plateau fracture (a) Axial CT scan view of the fracture. (b) An anteroposterior intraoperative radiograph following mini-open reduction of the articular surface and application of an Ilizarov frame with knee bridging. (c) Lateral view of the knee joint after the removal of the knee bridging. (d, e) Range of motion in extension and flexion six months postoperatively in the same patient.

Only one patient presented with pin track infection and was treated with local pin care and oral antibiotics without requiring removal of wires or half pins. Three patients presented with decreased range of knee motion due to stiffness and were treated with mobilization under anesthesia. Ilizarov external fixator was discharged after an average time of 4.4 months (range: 4 to 6 months) (Figure 3).

## Discussion

Complex tibial fractures are usually associated with knee stiffness and walking impairments [8]. Although there are more than 32 different classification systems, the Schatzker, AO/OTA, and recently the Luo three-column classification systems are vastly utilized [9]. In our opinion, no matter which classification is used, the preoperative CT scan is of great importance and the most useful tool for planning the surgical intervention.

Treatment modalities currently include closed reduction and casting, open reduction and internal fixation, limited internal fixation with external circular fixator, and a combination of an internal fixation with a hybrid external fixator [10]. The anatomical and clinical outcome seems to be equal in the treatment of tibial plateau fractures regardless of the treatment method [11]. There are studies suggesting that ring fixation system combined with minimal internal fixation provides better clinical outcome [10].

The external fixator as a definitive treatment for patients with complex tibial plateau has also been described [12]. Ηall et al. stated that good clinical outcomes could be achieved in complex fractures treated by external fixator than open reduction internal fixator [13]. Ligamentotaxis is used for reduction [14]. It is a term used to emphasize that for traction to be effective, it must be balanced by counter traction provided by ligaments and soft tissue surrounding the bone. Bridging across the knee increases the stability of the fixation, reduces the forces on the articular surface, and lessens the incidence of loosening of reduction in severely comminuted fractures [13]. We prefer joint stability rather than the potential stiffness due to the duration of bridging. Furthermore, the potential knee stiffness that can be seen postoperatively can provide stability for the fracture healing process [10]. Up to 90% results with good or excellent range of motion and stability are reported in patients who treated with Ilizarov external fixator as definitive treatment in complex tibial plateau fractures [15]. Keightley et al. reported good long-term healing and functional outcomes in Schatzker type IV-VI tibial plateau fractures treated with an Ilizarov technique [16,17]. Debnath et al. recommended the Ilizarov technique for Schatkzer type VI tibial plateau fractures with comminution [18]. Reddy et al. reported good healing rates and low incidence of infection in Schatzker type II and above tibial plateau fractures [19]. In our study, we have also evaluated Schatzker type II-VI tibial plateau fracture and found similar results.

Stable open reduction internal fixation and early mobilization for these injuries is the gold standard, but there is a significant risk. The extended incision to gain access to both medial and lateral plateau causes further soft tissue damage, increases the risk of infection, and delays union. Poor outcomes are related with severe soft tissue injury [20]. There is a list of potential complications ranging from early wound problems to amputation in case of a severe infection [10,14,21]. McNamara et al. also stated that there is not enough evidence to ascertain the best method of fixation [22].

One of the treatment goals in complex tibial plateau is the preservation of the soft tissue envelope bearing in mind a potential future total knee arthroplasty. Bove et al. suggest that Ilizarov frame is more effective than open reduction and internal fixation in complex tibial plateau fractures due to low incidence of infection and early mobilization [23]. In our study, only one (0.02%) patient had a pin track infection. Frames preserve the blood supply, protect the osteogenic factors, and permit early function of muscles, thus allowing early mobilization. They also allow potential change of fragment position in multiple planes with the fixator in place. Moreover, they permit fragments stabilization distally with the same device. Ring-tensioned wire frames provide mechanical stability of the fracture comparable with dual-plating internal fixation [24]. Barbary et al. reported a low rate of morbidity and satisfactory clinical outcome with the combination of Ilizarov technique and minimal internal fixation in patients with Schatzker type VI tibial plateau fractures [25].

A residual varus or valgus deformity is common. This could be attributed either to initial malreduction or postoperative loss of reduction. Nevertheless, moderate deformity is compatible with satisfactory clinical outcome [26]. Maintenance of mechanical axis is one of the goals of treatment and correlates with excellent results in function and clinical outcome. In our study, patients with joint alignment equal to or more than 5° presented statistically significant lower values of AKSS compared to those with lower than 5°. According to Rademakers et al., malalignment of the mechanical axis greater than 5° of the controlateral limb tripled the incidence of degenerative osteoarthritis (27% vs. 9%) at up to 27 years follow-up. Age at the time of injury had no effect on functional results [27].

There is a wide range of acceptable residual articular surface displacement ranging from 2 to 12 mm [26,28]. According to our study results, the postoperative articular surface depression showed a statistically significant correlation with the AKSS. Patients with articular depression > 4 mm had poor or fair results, whereas patients with articular depression < 4 mm had good or excellent results. The increase of articular surface impaction by 1 mm causes reduction of AKSS by 15 units. Based on the very high value of R2 of articular surface impaction variable in the multiple linear regression model, we can assume that the collapsed joint is the most important factor that affects the functional status of a patient (Table 5). Our findings are in accordance with the recent study by Kateros et al., who reported that patients who were treated with hybrid external fixator and had residual joint depression of > 4.5 mm showed significant poor or fair results with the AKSS [29].

Giannoudis et al. stated that multiple factors, such as knee stability, varus or valgus malalignment, and preservation of the meniscus, have a significant role in the clinical outcome and risk of posttraumatic osteoarthritis than articular congruity. They also reported that the thicker cartilage of the tibial plateau in relation to other joints potentially reduces the risk of posttraumatic osteoarthritis compared with similar articular depression at other joints [30]. To our knowledge, there are a few studies evaluating the articular surface reduction by using fluoroscopy intraoperatively. There is no clear evidence about the specificity, sensitivity, and interobserver agreement in grading the articular surface step-off using fluoroscopy intraoperatively or X-rays postoperatively. Preoperative CT is more useful than X-rays for the classification and evaluation of tibial plateau fractures. Postoperative CT scan is important in determining the reduction of the articular surface and the healing process [30]. Unfortunately, this is performed when the patient has already left the operating theater.

## Conclusions

Ilizarov external fixator with knee bridging and mini-open reduction as a definitive treatment option for Schatzker type II-VI tibial plateau fractures provides adequate stabilization and restores articular surface and mechanical axis without compromising the soft tissue envelope. The increase of articular surface impaction by 1 mm causes reduction of AKSS by 15 units. Patients with joint alignment equal to or more than 5° present lower values of AKSS. The preoperative CT scan is of great importance and the most useful tool for planning surgical intervention no matter which classification system is used.
